# 

**Published:** 1810-11

**Authors:** 


### MONTHLY CATALOGUE.

Description of an Affection of the Tibia induced by Fever; with Observations on the Treatment of this Complaint. By Thomas Whately, Surgeon. 8vo. London, Callow.

A Dissertation on Retroversion of the Womb, including some Observations on Extra-uterine Gestation. By Samuel Merriman M. D. 8vo. Lond, Callow.

A Practical Treatise on the Morbid Sensibility of the Eye, commonly called Weakness of Sight. By John Stevenson. 8vo, Lond. Callow.

A Commentary on the Treatment of Ruptures, particularly in a State of Strangulation. By Edward Geoghegan. Lond.

Examinations in Anatomy, Physiology, Practice of Physic, Surgery, Materia Medica, Chemistry and Pharmacy; for the Use of Students who are about to pass the College of Surgeons, or the Medical and Transport Boards. By R. Hooper, M. D. 12mo. Lond.

The Rabies Piratica, its History, Symptoms and Cure; also the Furor Hippocraticus, or Græco-mania, with its Treatment. By Bryan Crowther. 8vo. Lond.

The Apollo; or Medical Censor, &c. published weekly, in Numbers, No. I. published on the 29th of September last. 8vo. Lond. Crippes,

A General Catalogue of an Extensive Collection of Books in Medicine, Surgery, Anatomy, Chemistry, Midwifery, Materia-Medica, Botany, Farriery, &c. By T. Underwood, West Smithfield.

This Catalogue has attached to it, a useful Appendix, containing Tables of the pay of the Medical Departments of the Army, Navy, and East India Company; and a copleat List of the Lectures delivered in London, with their Terms, Hours of Attendance, &c.

A New Edition, much improved and enlarged, of Fyfe's Compendium of Anatomy. 3 vols 8vo. with plates, Underwood.

Transactions of the Medical Society of London. Vol. I. Part I. 8vo.

A Catalogue of a Modern Collection of Books in Anatomy, Medicine, Surgery, Chemistry, Botany, &c. Part I. with an Appendix containing an extensive Catalogue of Medical Publications, in the year 1810. By J. Callow, Crown Court.

The Second Part, containing a large Collection of second-hand Medical Books in various languages will be spedily published.

Advice to such Military officers and others, who may be suffering from what has been called the *Walcheren Fever*. By Charles Griffiths, M. D, 8vo. Callow.

Examination

Examination of the Prejudices commonly entertained against Mercury, as beneficially applicable to the greater number of Liver Complaints, and to various other Forms of Disease, as well as to Syphilis. By James Curry, M. D. F. R. S. Physician to Guy's Hospital. 8vo. Callow and Cox

---

### TO CORRESPONDENTS.

We take the liberty of remarking to our ingenious and acute correspondent, *Joshua Sylvestre*, that the palpable hiatus in the title page of his poetical progenitor's attack on Tobacco, as he quotes it, has probably arisen from his citing from memory. In the folio edition, which now lies before us, where the "*Tobacco Battered*" is printed with Sylvestre's translation of *Du Bartas*, it runs thus.

TOBACCO BATTERED,
and
The Pipes shattered about their ears, that idlely
idolize so base and barbarous a
WEED;
Or at least-wise over-love
so loathsome Vanitie.

Various communications are under consideration, some of which are intended for insertion in the next number.

We must observe to our angry correspondent AMICUS, that there is not much of equity in his determination to visit upon us the sins of our predecessors, if sins they had. With regard to the particular subject of his complaint, we can reply, that we had no concern in the Journals in which the promise was made. We see, however, a propriety in ending the first series of the MEDICAL and PHYSICAL JOURNAL with the 30th volume, which never did apply to the 24th. When that time comes, and which is not very distant, we assure *Amicus*, and, as we have never broken our word with him, or with the public, we expect he will give us credit for the assurance, that a full and correct GENERAL INDEX will appear; pointing out distinctly the multifarious materials of this elaborate work, collected during a most interesting period of MEDICAL SCIENCE.

